# MRI tracking stem cells transplantation for coronary heart disease

**DOI:** 10.12669/pjms.304.4936

**Published:** 2014

**Authors:** Xi Lu, Rui Xia, Bing Zhang, Fabao Gao

**Affiliations:** 1 Xi Lu, Molecular Imaging Laboratory, Department of Radiology, West China Hospital, Sichuan University, No.1, Ke Yuan Road 4, Gao Xin District, Chengdu, 610041, Sichuan, China.; 2 Rui Xia, Molecular Imaging Laboratory, Department of Radiology, West China Hospital, Sichuan University, No.1, Ke Yuan Road 4, Gao Xin District, Chengdu, 610041, Sichuan, China.; 3 Bing Zhang, Molecular Imaging Laboratory, Department of Radiology, West China Hospital, Sichuan University, No.1, Ke Yuan Road 4, Gao Xin District, Chengdu, 610041, Sichuan, China.; 4 Fabao Gao, Molecular Imaging Laboratory, Department of Radiology, West China Hospital, Sichuan University, No.1, Ke Yuan Road 4, Gao Xin District, Chengdu, 610041, Sichuan, China.

**Keywords:** Stem Cell Transplantation, Magnetic Resonance Imaging, Coronary Disease

## Abstract

Cardiovascular disease is the leading cause of mortality worldwide. Stem cell transplantation has become a new treatment option for cardiovascular disease because the stem cells are able to migrate to damaged cardiac tissue, repair the myocardial infarction area and ultimately reduce the role of the infarct-related mortality. Cardiac magnetic resonance imaging (MRI) is a new robust non-invasive imaging technique that can detect anatomical information and myocardial dysfunction, study the mechanism of stem cells therapy with superb spatial/temporal resolution, relatively safe contrast material and lack of radiation. This review describes the advantages and disadvantages of cardiac MRI applied in stem cells transplantation and discusses how to translate this technique into clinical therapy.

***Sources of Data/Study Selection:*** Data from cross-sectional and prospective studies published between the years 2001-2013 on the topic were included. Data searches included both human and animal studies.

***Data Extraction:*** The data was extracted from online resources of statistic reports, Pub med, THE MEDLINE, Google Scholar, Medical and Radiological journals.

***Conclusion:*** MRI is an appealing technique for cell trafficking depicting engraftment, differentiation and survival.

## INTRODUCTION

Cardiovascular disease is a worldwide leading cause of mortality. In recent years interventional and surgical treatment of coronary heart disease using coronary artery bypass grafting (CABG) has made great progress. However, it’s mortality rate is still as high as 13%, while the five-year prevalence of heart failure remains as high as 50%.^1^ The current conventional treatment methods are unable to prevent the necrosis of myocardial tissue and cannot restore the function of infarcted myocardium. Currently, stem cell research has achieved many breakthroughs in transplantation therapy and its feasibility and effectiveness has been demonstrated in animal experiments as well as in small-scale clinical trials. 

For myocardial infarction stem cell transplantation therapy, many issues remain unresolved: How are the optimal number of cells determined when performing cell transplantation therapy? How is the survival time of transplanted cells best monitored? Do the transplanted cells undergo differentiation (e.g. into cardiomyocytes, smooth muscle cells or endothelial cells)? Can transplanted cells produce electrochemical coupling of normal myocardial and cardiac cells and their functions change? What is the mechanism of cells transplantation in the treatment of myocardial perfusion and cardiac function after a short enhancement (myocardial cell regeneration or paracrine or other)? These are examples of the current key issues that cardiac stem cell therapy research must address.^2^

 Imaging methods allow us to trace and track stem cells in vivo and better evaluate the efficacy of cell therapy. Commonly, imaging methods in current usage include ultrasound imaging, PET/SPET, magnetic resonance imaging (MRI), optical imaging and CT imaging. Among them, cardiac MRI is undoubtedly the most promising molecular imaging means in clinical transformation as it can provide anatomical information with relative safety and superior resolution and sensitivity without radiation.^3^^,^^4^

***Stem cell transplantation treatment: ***Stem cell transplantation methods using adult stem cells, embryonic stem cells（ESC）or induced pluripotent stem cells (iPSC) have become a major research focus in the field of treatments for ischemic heart disease. For example, using an MRI detection method, Grajek and colleagues have reported that patients with acute anterior wall myocardial infarction show improved myocardial perfusion 12 months after injection of bone marrow stem cells (BMCs), but that the left ventricular ejection fraction (LVEF) does not increase.^5^ Kraehenbuehl and colleagues have also reported that embryonic stem cell transplantation in a rat myocardial ischemia model can reduce the left ventricular expansion and the area of myocardial infarction after 3-6 weeks.^6^ Furthermore, Nelson and colleagues have shown that treatment with human stem cell factor, OCT3/4, SOX2, KLF4 and c-MYC is able to induce embryonic stem cells into iPSCs, form regenerative myocardial, smooth muscle or endothelial vascular cells in situ, repair myocardial infarction and increase ventricular wall thickness and electrical stability.^7^ In addition, a meta-analysis of randomised controlled clinical trials demonstrated that intracoronary adult bone marrow stem cell therapy in the setting of acute myocardial infarction (AMI) could improve left ventricular function and reduce the incidence of heart failure.^8^ Table-I shows the major cell types commonly used for autograft or allograft transplantation in myocardial repair.

***Direct labelling of stem cells: ***It is difficult to produce ideal stem cell MRI contrast effects. Hence, a great deal of research into the MRI-tracing of transplanted stem cells has focused on the development and application of novel contrast agents. Commonly used stem cell MRI contrast agents are divided into two major categories. The first involves contrast agents based on gadolinium (Gd) and manganese (Mn), such as gadolinium chelating agents (Gd-DTPA)^9^ and manganese chloride (MnCl_2_).^10^ These mainly provide T1 positive contrast effects. The second involves paramagnetic/super paramagnetic contrast agents based on iron oxide nanoparticles that produce strong T2/T2* negative contrast effects.^11^^,^^12^

***Strategies of iron oxide particle labelling: ***Iron oxide nanoparticles are a family of paramagnetic/superparamagnetic contrast agents, consisting of a ferrite (maghemite or magnetite) core and a polymeric coating. Depending on the diameter sizes (including both metal core and polymeric coating), the nanoparticles can be divided into the SPIO (diameter size 60nm-150nm), USPIO (diameter size 10nm-40nm) and MION (diameter size 10nm-30nm) categories.^13^ Ferucarbotran (Resovist) and Ferumoxides (Endorem or Feridex) are MRI enhancement contrast agents approved by FDA, that have been widely applied in the clinical diagnosis of liver tumours and metastatic lymph nodes. High concentrations of ferromagnetic material can shorten both the T1/T2 values as well as the effect of T2*, resulting in a significant reduction in MR relaxation and higher biological safety.^14^^-^^16^

**Table-I:** Different cell types of cell transplantation for myocardial repair

***Allogenic sources stem cells***	***Autologous sources stem cells*** ***(Adult stem cell)***
Embryonic stem cells Foetal cardiomyocytes Human umbilical cord-derived cells	Resident cardiac stem cells Adipose-derived stem cells Skeletal myoblasts Bone marrow-derived Mononuclear/CD34^+^ fraction Mesenchymal stem cells Endothelial progenitor cells Multipotent adult progenitor cell Induced Pluripotent Stem cells

Most labelled cells do not spontaneously internalise SPIOs and effective strategies are therefore needed to promote endocytosis. For example, positively charged polymer transfection agents(TAs) can be coated on the surface of magnetic iron oxide particles resulting in negatively charged cells nonspecifically uptaking the particles through the membrane surface. At present, composites of SPIO and polycation TAs are the most commonly used method to enhance the endocytosis of iron oxide particles.^17^^-^^19^ Due to the negatively charged membrane surface, the unmodified ferric oxide particles are unable to attach to the stem cells. Polycation TAs are macromolecular substances with positively charged surfaces that may include polylysine^20^ or protamine sulfate.^18^ With a strong positive/negative interaction, SPIO/TA composites are able to adhere to the surface of cell membranes, improve the phagocytosis of iron oxide particles and avoid aggregation of SPIO particles.^21^ For example, Frank and colleagues mixed Ferumoxides (Feridex) with USPIO (MION-46 L) and added cationic TAs, successfully raising the concentration of intracellular SPIO particles. After 4-48 h incubation with 25 µg Fe/ml TA-(USPIO), target cells demonstrated a significant reduction in T2 values.^22^^,^^23^ Subsequent work has optimized this method by mixing Ferumoxides with protamine sulphate (50:3) µg/ml. Following incubation with the human mesenchymal stem cells, hematopoietic CD34^+^ stem cells and other mammalian cells overnight, the iron content of the cells was found to be 1.47 pg/cell-17.31 pg/cell.^24^

Besides cationic polymerization material coating, other iron oxide particle surface modifications can also enhance cellular endocytosis. Koyama and colleagues fused monoclonal antibodies of pancreatic cancer specific antigen (PAP2a) with dextran modified SPIOs. Due to the nature of antigen-antibody reactions, this represented a novel approach for targeting the iron oxide particles to pancreatic cancer cells and promoting receptor-mediated SPIO endocytosis.^25^ In addition, iron oxide particle surfaces can be modified by receptors such as vascular cell adhesion molecule-1^26^ and membrane mucin A5^27^ resulting in the nanoparticle targeting of specific tissues or organs. These methods require specific target receptors in the membrane, which greatly limits their application in stem cell tracking. Another method for increasing the efficiency of nanoparticle endocytosis is known as magnetoelectric perforation. Toxicity testing of mesenchymal stem cells, neural stem cells and adipose cells in vitro have all produced encouraging results with this method. Magnetoelectric perforation does not require a prolonged cell incubation time and the contrast agent for the target cells is safe and effective and approved by the FDA.^28^^,^^29^ However, from the perspective of biological safety considerations further research is still needed.

***Biological safety of iron oxide particle labelling: ***Cell labelling with iron oxide provides the potential for a rapid translation from preclinical medicine to a clinical setting. However, intensive toxicity tests are necessary for every protocol and cell type before clinical application. SPIO agents approved by the FDA (Feridex, Resovist and Endorem) are mainly cleared by the reticuloendothelial system. Richards and colleagues isolated peripheral blood mononuclear cells from volunteers, labelled them with Ferumoxides in vitro and administrated these cells through intravenous injection. In T2* weighted images and R2* maps they observed that the labelled cells could efficiently migrate to the inflammation damage areas in tuberculin skin test.^15^ In spite of this result, many additional pre-clinical experiments will be required to verify the bio-safety of paramagnetic contrast agents. 

***Sensitivity of MRI detection in vivo:*** The MRI detection of labelled stem cells is affected by intracellular iron distribution, MRI sequence, spatial resolution, magnetic field intensity and surrounding tissue heterogeneity. A higher intracellular iron content results in a more obvious relaxation time shorten. The T2* weighted image is highly sensitive for iron oxide particle labelling and its sensitivity can reach 3000 times that of T1 WI or 60 times that of T2 WI. ^30^ The most commonly used T2* sequence is the steady-state free precession (SSFP) and it is now the preferred sequence for detecting SPIO labelled cells. The T2* sequence is vulnerable to the influence of intracellular magnetic field inhomogeneity and the interference of the surrounding normal tissues, especially in high field MRI. However, conventional fast 3D gradient echo (GE) sequences are able to balance T2* sensitivity, spatial resolution and imaging time. In 2006, Fayad and colleagues reported an appealing MR sequence named ‘Gradient echo Acquisition for Superparamagnetic particles with Positive contrast’ (GRASP), which creates a positive rather than negative contrast of SPIO. The main advantage of this method is that it overcomes the interference of other sources of T2* effects and that the hyperintense signal may increase the sensitivity and specificity of cell tracking.^31^ Theoretically, when the quantity of the target cells is small, a smaller imaging voxel size should be chosen in the high field MRI. 

***Limitations of SPIO direct labelling: ***Although the SPIO particle direct tracing method is the most commonly used cell labelling technique for MRI, it still has some shortcomings. For example, signal is usually found in areas of noninterest (e.g. the cardiopulmonary junction).^32^ Also, as a paramagnetic material accumulating in haemorrhagic infarction disease, haemoglobin shows low signal on T2* weighted imaging.^33^ It is important to note that with the death and rupture of transplanted cells, targeted SPIO nanoparticles can be phagocytosed by surrounding tissue cells or reticuloendothelial cells and subsequently redistributed, deposited or differentiated. Considering these facts, direct iron oxide labelling is more appropriate for short-term cell tracing in vivo or in vitro. The reason for false positive signals may be due to phagocytosis by surrounding cells, such as macrophages, or simply iron oxide distributed extracellularly. Moreover, partial volume effects or low concentrations of cells in one imaging voxel can lead to false negative results and with every subsequent cell division intracellular iron content will be halved, thus leading to a gradual reduction in cell detection sensitivity. For example, it has been reported that after 6 weeks MRI is unable to detect the differences between visible and invisible cells after transplant stem cell were administrated to the heart.^34^ Despite these limitations and shortcomings, paramagnetic/superparamagnetic iron oxide particles are still highly popular in the field of stem cell tracing largely because of their high sensitivity.

***Reporter gene labelling: ***Reporter gene labelling is achieved through the fusion of an MRI reporter gene to a target gene. Transfection of a target cell can then produce reporter gene expression for indirect MRI detection in vivo. The products of reporter genes are only expressed in living cells because genes are incorporated into the cellular DNA via transgenic methods. Thus, with these important advantages, transgenic gene labelling strategies are highly valuable in long-term studies of labelled cell survival, proliferation and differentiation in vivo. 

The MRI reporter gene can be divided into two categories based on the product of its expression: 1) Intracellular enzymes ^35^ including β-galactosidase, cytosine deaminase, creatinine kinase, tyrosinase and arginine kinase; 2) Ferritin or transferrin receptors.^36^ Among MRI reporter genes, the ferritin receptor has attracted a great deal of attention. Ferritin is a metalloprotein composed of 24 subunits that can bind up to 4500 Fe^3+^ ions. Excessive expression of ferritin can increase the intake of iron and the redistribution of intracellular iron which leads to the accumulation of transverse relaxation rates and a reduction of T2 values. Genove and colleagues reported that following adenovirus-ferritin reporter gene injection into murine corpus striatum, a robust contrast could be observed on T2 and T2* weighted imaging from 5 to 39 days.^37^

Despite the advantages of MRI reporter gene imaging, there still remains a difficulty in avoiding potential damage to cell proliferation and differentiation. Additionally, apart from concerns regarding the sources and safety of cells, issues relating to gene mutation and sensitivity still need to be solved.

## CONCLUSION

The rapid development of in vivo imaging techniques has benefited the dynamic monitoring of stem cell therapies following myocardial infarction. CMR, which is safe, sensitive, lacks radiation and provides good resolution can produce accurate information regarding anatomy and changes in cardiac function. Currently, CMR is undoubtedly the most likely methodology regarding the clinical prospects of molecular imaging technologies. For researchers who study MRI, a major aim of future work is to develop new molecular probes and sequences in order to improve its sensitivity and specificity. At the same time the potential biological damage caused by reporter genes and immune responses needs to be limited. With the help of imaging technology, stem cell therapy will have a major part to play in the therapy of myocardial infarction.

## Authors Contribution:

***Xi Lu, Rui Xia, and Bing Zhang: ***Carried out the literature search and wrote the manuscript.

***Fabao Gao:*** Reviewed and finalized the article.
